# National approaches to the vaccination of recently arrived migrants in Europe: A comparative policy analysis across 32 European countries

**DOI:** 10.1016/j.tmaid.2018.10.011

**Published:** 2019

**Authors:** Sofanne J. Ravensbergen, Laura B. Nellums, Sally Hargreaves, Ymkje Stienstra, Jon S. Friedland, ESGITM Working Group on Vaccination in Migrants

**Affiliations:** a Department of Internal Medicine/Infectious Diseases, University of Groningen, University Medical Center Groningen, Groningen, the Netherlands; b Institute for Infection and Immunity, St George's, University of London, London, WC1E 7HU, UK; c Section of Infectious Diseases & Immunity, Imperial College London, London, UK

**Keywords:** Vaccine preventable diseases, Immunisation, Vaccination, Migrants, Refugees, European Union, Health policy

## Abstract

**Background:**

Migrants may be underimmunised and at higher risk of vaccine-preventable diseases, yet there has been no comprehensive examination of what policies are currently implemented across Europe targeting child and adult migrants. We analysed vaccination policies for migrants in 32 EU/EEA countries and Switzerland.

**Methods:**

Using framework analysis, we did a comparative analysis of national policies and guidelines pertaining to vaccination in recently arrived migrants through a systematic guideline and literature review and by approaching national experts.

**Results:**

Six (18.8%) of 32 countries had comprehensive policies specific to the vaccination of migrants (two focused only on child migrants, four on both adults and children). Nineteen (59.4%) countries applied their national vaccination schedule for migrant vaccinations, predominantly focusing on children; and five (15.6%) countries had circulated additional migrant-specific resources to relevant health-care providers. In six (18.8%) countries, policies on migrant vaccination focused on outbreak-specific vaccines only. In ten (31.3%) countries, policies focused on priority vaccinations, with polio being the vaccine most commonly administered and heterogeneity noted in vaccines recommended to adults, adolescents, and children. Eighteen (56.3%) countries recommended that an individual should be considered as unvaccinated where vaccination records were missing, and vaccines re-administered. Nine (28.1%) countries reported that specific vaccinations were mandatory.

**Conclusion:**

There is considerable variation in policies across Europe regarding approaches to vaccination in adult and child migrants, and a lack of clarity on optimum ways forward, what vaccines to offer, with a need for robust research in this area. More emphasis must be placed on ensuring migrant-specific guidance is disseminated to front-line healthcare professionals to improve vaccine delivery and uptake in diverse migration populations across the region.

## 1 Introduction

Migrants within the European Union (EU) may represent an under-immunised group, with implications for outbreaks of vaccine-preventable diseases [1]. Outbreaks of measles and hepatitis A have been documented in migrant populations in Europe [2,3], and diseases including poliomyelitis remain endemic in some migrant sending countries [4]. Migrants in the EU and European Economic Erea (EEA) are a diverse group, including both internal EU migrants – moving from one country in Europe to another – and external non-EU migrants. Although the role of migrants in epidemics of vaccine-preventable diseases is unclear, mainly due to poor data collection in this area, the current multi-country measles epidemic in the EU/EEA has involved EU migrants moving from and between countries with large epidemics [5]. Large numbers of recently arrived migrants to the EU may have an uncertain vaccination status, including incomplete vaccination history and/or missing documentation of previous vaccinations, with implications for health-care providers and how to approach catch-up vaccination [6]. In a cohort of 2126 asylum-seeking children to Denmark 30% were considered not to be immunised in accordance with the Danish schedule, with underimmunisation particularly high in adolescent migrants (aged 10–17 years) [7]. Strategies and approaches to engaging migrant populations in vaccination are not clear due to the lack of high quality studies assessing vaccination implementation [8].

A recent report has highlighted wide disparities in access to healthcare and vaccination across Europe, with undocumented (irregular migrants) in particular unable to access free vaccination because of administrative barriers and lack of entitlement to free health services including vaccination services [9]. This is despite the fact that ensuring high levels of coverage is a key priority of the European Vaccine Action Plan [10], in which all countries have committed to eliminating endemic measles and rubella (>95% coverage with the measles mumps rubella vaccine), controlling hepatitis B infection, and sustaining polio-free status. Innovations in service provision to ensure hard-to-reach groups, including migrants, access vaccination services remains an important component to reducing vaccine-preventable diseases in Europe.

However, current approaches to the vaccination of migrants have not been well documented to date, and it is acknowledged that there are additional challenges in ensuring equitable access to vaccines in diverse and mobile migrant populations [9,11]. The ongoing refugee crisis has facilitated renewed dialogue around approaches to the screening and vaccination of recently arrived migrants for infectious diseases. The World Health Organization (WHO), United Nations High Commissioner for Refugees, and the United Nations Children's Fund recommended in 2015 that migrants in the WHO European Region should be vaccinated soon after arrival in accordance with the immunisation schedule of the receiving country in which they intend to stay for more than a week [11], and the European Centre for Disease Prevention and Control (ECDC) is currently developing guidance on approaches to vaccine-preventable diseases in newly arrived migrants [12]. However, there has to date been no comprehensive examination of what policies or guidelines are currently implemented across Europe, or how they compare across countries. In order to facilitate the harmonisation of vaccination policies across Europe and identify best practice, a clear understanding of the different policies and of the key gaps or inconsistencies in such policies is needed [13,14]. We therefore did a comparative analysis of policies and guidelines in EU/EEA countries and Switzerland relating to the provision of vaccinations to recently arrived migrants to identify common approached.

## 2 Methods

We documented and analysed vaccination policies for migrants in 32 EU/EEA countries, and Switzerland. The policy analysis was guided by Bardach's health policy framework [15,16], and consisted of a comparative analysis of policies or guidance for vaccination in migrants across European countries. Primary and secondary data sources were used to identify evidence for the analysis. Key migrant groups included recently arrived migrants (foreign born, in the host country <10 years), refugees (granted asylum), asylum seekers (awaiting a decision on their asylum application in the host country), and undocumented migrants (without necessary authorisation or documents required under host country's immigration regulations). Primary data were obtained through contacting national experts in each country, who were asked to provide both relevant health policy documents. Secondary data consisted of relevant health policy documents and guidelines around vaccination in migrants, which were obtained through a systematic search of the literature and published papers from relevant health bodies such as Ministries of Health.

### 2.1 Approaching national experts

National experts for the included EU/EEA countries and Switzerland were identified through the network of the European Study Group for Infections in Travellers and Migrants (ESGITM), which contributes to activities in the field of travel and migration related infectious diseases as part of the European Society of Clinical Microbiology and Infectious Diseases (ESCMID). Experts were also identified through relevant publications and key meetings over the last 5 years (e.g. the European Congress of Clinical Microbiology and Infectious Diseases [ECCMID]). Between December 2016 and May 2017, we emailed experts in the following 32 countries: Austria, Belgium, Bulgaria, Cyprus, Croatia, Czech Republic, Denmark, Estonia, Finland, France, Germany, Greece, Hungary, Iceland, Ireland, Italy, Latvia, Liechtenstein, Lithuania, Luxembourg, Malta, the Netherlands, Norway, Poland, Portugal, Romania, Slovakia, Slovenia, Spain, Sweden, Switzerland and the United Kingdom (UK). National experts were asked to provide documents relating to national policies or guidelines on vaccination in migrants, with a key focus on national guidelines and definitions, mandatory vaccinations, outbreak specific guidance, priority vaccinations, and incomplete vaccination records. Non-respondents were contacted either by e-mail or phone twice in an effort to include all 32 countries. Where documents were provided in a language other than English, they were translated as needed.

### 2.2 Secondary data collection and analysis

Expert input was complemented by the identification of secondary data on health policies or guidelines across European countries through a systematic search of the literature. First, we searched PubMed and Google Scholar using terms relevant to migrants and vaccinations, including “vaccination”, “immunisation”, “vaccine preventable diseases”, “immunisation”, “migrants”, “refugees”, “European Union”, and “health policy” between inception and June 20, 2017. Additional literature reporting policies or guidance on vaccination in migrants was also identified through internet searches using relevant terms for each specific country, and hand searching through health policy documents and relevant national policy websites (e.g. for Ministries of Health).

### 2.3 Data analysis

Once the relevant data sources had been collated, we utilised framework analysis to synthesise relevant content on policies or guidance for vaccination in migrants across the included countries. Our policy analysis framework focused on key topics including national guidelines and definitions, mandatory vaccinations, outbreak specific guidance, priority vaccinations, and catch-up vaccination in the absence of complete vaccination records. Relevant policies or guidance were extracted and analysed for each country for each of the key framework themes.

## 3 Results

### 3.1 National guidelines and definitions

We identified guidance and policy documents for all 32 countries through our database, internet, and hand searches and received responses from experts in 30 EU/EEA countries regarding national or regional guidelines on vaccination in migrants. Using this two-pronged approach, we therefore collated policies and guidance from all 32 countries under study.

We identified significant variation in policies and guidelines for vaccination in migrants across the EU/EEA (Table 1). Six (18.8%) of 32 countries had specific vaccination guidelines for migrants, two of which applied only to child migrants and four to both child and adult migrants; we found that some of these guidelines were very comprehensive in terms of approaches to catch-up vaccination in adults and child migrants, and other were not. Five (15.6%) of 32 countries had circulated migrant-specific resources to relevant health-care providers. 19 (59.4%) of 32 countries apply their national vaccination plan for vaccination in migrants, and two (6.3%) countries used the International Organization for Migration (IOM) handbook with recommendations for vaccination in migrants [17].

**Table 1:** Policy and guidance on vaccination in migrants in EU/EEA countries.

Policy/guidance on vaccination in migrants	Number of countries [n = 32] (%)
Specific national policy/guidance for migrant vaccination	6 (18.8%) (2 for child migrants only; 4 for adult and child migrants)
Migrant-specific resources are available for healthcare workers; but no national policy/guidelines	5 (15.6%)
National vaccination plan is used and/or “catch up” vaccination document is circulated	19 (59.4%)
IOM handbook [17]	2 (6.3%)

### 3.2 Administration of vaccinations to adults, adolescents, and children

**Fig. 1:**
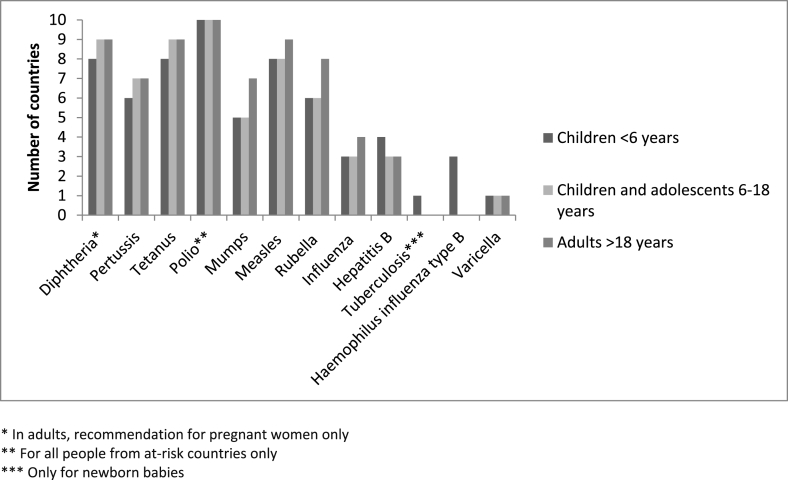
Priority vaccines across EU/EEA countries for migrant children, adolescents, and adults in those countries reporting migrant-specific policies.

There was considerable heterogeneity across countries regarding which vaccines should be administered to adults and child migrants. Ten (31.3%) countries in total had national guidance or which stated that specific vaccinations should be prioritised for migrants (Fig. 1). Polio was the most frequently reported priority vaccine given to recently arrived migrants, but vaccination for hepatitis B – for example – was not being considered. Fig. 1 highlights that there was considerable heterogeneity between what vaccines are recommended to adult, adolescent, and child migrants.

### 3.3 Outbreak-specific recommendations

Six (18.8%) countries reported having specific guidelines or policies in place regarding outbreak-specific vaccinations for migrants. These guidelines differed in content, but in general provided information to health-care providers in recognising certain infectious or epidemic diseases and a name to contact in case of an outbreak among refugees/migrants. One country recommended meningococcal vaccination when an outbreak is identified through the Ministry of Health's surveillance system [18,19]. In another country, a mobile medical team is responsible in certain areas for administering immunisations to adult migrants who have not been vaccinated in the case of an infectious disease outbreak [20].

### 3.4 Uncertain vaccination status

In 18 (56.3%) of 32 countries policies, guidelines, and/or resources stated that a person should be considered as unvaccinated where vaccination records are missing. In five countries a catch-up immunisation document was available to guide vaccination in migrants with missed vaccinations or a lack of records [[20], [21], [22], [23], [24]]. However, the approach to determine vaccination status varied across countries. For example, oral reporting of vaccination in children was not accepted as proof of vaccination status in most countries. In one country oral reporting of vaccination status was considered to be sufficient, and where there was uncertainty about the vaccination status children are then considered unvaccinated [25,26]. In another country, children below 5 years of age for whom vaccination records were lacking, are assumed to be unvaccinated and subsequently included in the childhood vaccination programme using vaccination intervals based on the age of the child. In this same country for children aged 5–17 years, DTaP-IPV/Hib primary vaccine (DiTeKiPol/Act-Hib) is administered once then antibodies against diphtheria and tetanus are measured 1 month later [27]. Specific guidance exists regarding incomplete vaccinations for migrants from the top three migrant-sending countries at the current time (Syria, Iraq, and Afghanistan) where vaccination coverage was relatively high prior to conflict but has since dropped [28]. This guidance recommends catch-up vaccination in children born since conflicts began in these countries, with reference to the likelihood that vaccination status may also be incomplete in adults arriving from these countries and for whom catch-up vaccination should also be considered [28]. For most countries, there is no specific guidance available on how to approach catch-up vaccination in adult migrants of uncertain vaccination status, but information on priority vaccines in adult migrants was available for 10 countries (Fig. 1).

### 3.5 Serology testing prior to vaccination

Four (12.5%) countries recommend against serology testing prior to vaccination for migrants with incomplete vaccinations or a lack of documentation on previous vaccination [22,[24], [25], [26], [27], [28], [29], [30], [31]]. The main reasons noted in the guidance against serology testing included: that the interpretation of serology tests is difficult (3 countries), serology is expensive (2 countries), false negative results often occur (1 country), and that migrants can easily and safely be revaccinated (1 country). Two (6.3%) countries, however, do recommend serology testing prior to vaccination. For example, the guideline recommends hepatitis B serology testing at the health check on arrival to the country, performed as part of screening for these infectious diseases [32]. Another country recommended the approach that all migrant women of childbearing age without a history of varicella infection should have their immunity checked, and that women with negative serology should be vaccinated [33].

### 3.6 Mandatory vaccination

We found considerable variations across EU/EEA countries in relation to whether vaccinations were mandatory or voluntary for migrants, with nine (28.1%) countries reporting that specific vaccinations were mandatory according to national policy. The definition and regulation of ‘mandatory’ vaccination policies were not well described, nor were consequences if vaccinations were refused. In most countries, guidelines did not stipulate that vaccinations were mandatory, though they were considered highly recommended.

## 4 Discussion

### 4.1 Summary of key findings

There were striking variations in terms of policy and guidance regarding vaccination in migrants across EU/EEA countries, with six (18.8%) of 32 countries having comprehensive policies specific to the vaccination of migrants, of which 2 focused only on child migrants. More than half of the countries applied their national vaccination schedule for migrant vaccinations, which is a response advocated by WHO and others [13]. In ten (31.3%) countries, policies focused on priority vaccinations, with polio being the vaccine most commonly administered and heterogeneity in which vaccines were recommended to adults, adolescents, and children. Some countries reported migrant-specific guidelines relating to outbreak-specific vaccine-preventable diseases only, and substantial variation was found across countries relating to whether vaccinations were mandatory. Our analysis found differences across countries when migrant presented to a health service with a missing or incomplete vaccination record, a common phenomenon in this group, with eighteen (56.3%) countries recommending that an individual should be considered as unvaccinated where vaccination records were missing, and vaccines re-administered.

### 4.2 Strengths and limitations

We aimed to provide a comprehensive examination of policies and guidance on the vaccination of recently arrived migrants in EU/EEA countries and Switzerland. Whilst we aimed to systematically search the secondary literature as well as contact national experts in this field in each European country, the field of vaccination among migrants is moving quickly and policies and guidelines are dependent on political context. Our policy, guideline, and literature search was done up to May 2017, and we are aware that more guidelines could have been published and circulated since this date; however, we have been in dialogue with vaccination experts since May 2017 and are not aware of anything significant that has been published and that would change our overall key findings. There are also numerous regional or local level policies or guidelines that may be implemented across Europe, as well as unpublished documents, which were not identified through our research.

### 4.3 Policy versus practice

It is not clear to what extent these policies, guidelines, and circulated resources were implemented in practice. In a recent EU-EEA-wide questionnaire survey, we found that implementation is considered by experts to be poor, with few initiatives targeting migrants specifically, and that adult migrants may be particularly excluded from catch-up vaccination on or after arrival [34]. This survey also highlighted a lack of clarity around what vaccinations should be given to adult and child migrants. These shortfalls have been reported by others, noting that high quality studies assessing vaccination implementation in migrant populations are lacking with which to inform policy making in this area [8].

### 4.4 Current shortfalls and next steps

The lack of national guidance around provision of basic care to recently arrived migrants has been previously reported [35], and it is well known that approaches to screening for infectious diseases in migrants varies considerably across the EU/EEA [36]. Migrants, we know, face multiple barriers to accessing healthcare on arrival to a host country, including vaccinations. It may be that there is a need for harmonisation of migrant vaccination approaches across European transit and receiving countries, guided by existing recommendations such as those produced by the IOM and others [17,35,[37], [38], [39]], involving non-governmental organizations and statutory service providers, and including some mechanism for monitoring and recording the delivery and uptake of vaccines to migrants specifically. This was highlighted by the European Parliament in January 2016, who called for health policies and health systems in the WHO European Region to better acknowledge migrants [39]. In light of increasingly restrictive health policies across Europe [40] vaccinations must be provided free of charge to high-risk groups, which aligns with European and international recognition of migrants’ rights to health [41]. There are clear clinical, public health, and human rights arguments for promoting access to an acceptable level of free health care, including vaccination, to migrants.

We report that vaccinations were mandatory for migrants in eight (26.7%) countries, a finding supported by data from the Vaccine European New Integrated Collaborative Effort (the VENICE project) – initiated to improve and monitor vaccination programmes in Europe [42]. VENICE data shows that compliance to vaccination programmes in European countries without mandatory vaccination is high among migrants and non-migrants [42], and they question to what extent vaccinations should be mandatory, suggesting that this is an area that needs warrants further discussion. There are numerous reasons migrants may wish not to be vaccinated e.g. cultural beliefs and legal reasons, such as seeking to avoid registration in a country which may require them to claim asylum in that country [13] as well as significant barriers to accessing care, and more robust research is needed to elucidate the key concerns of migrants around vaccination uptake on arrival to an EU/EEA country.

The ECDC vaccine schedule database allows comparison of vaccination policies between countries, highlighting immunisation schedules in all EU countries [43], but the database does not have data on vaccine schedules for migrants specifically. More emphasis must be placed on improving data collection around vaccine-preventable diseases in migrants in the EU/EEA, to better understand the extent to which these groups are both underimmunised and involved in outbreaks, so that targeted programmes can be implemented in relevant groups. The priority vaccines reported in guidelines in this policy analysis reflect current ECDC recommendations that vaccinations be offered according to national immunisation guidelines, with priority given to easily transmitted and/or serious infectious diseases such as polio [38]. The Canadian evidence-based guidelines on the vaccination of newly arrived migrants is more extensive – and covers both adult and child migrants – giving priority to MMR, DTP, polio, varicella, hepatitis B and tuberculosis. We have presented these guidelines in Table 2 to give a clear overview of what EU/EEA countries could adopt in terms of catch-up vaccination [37]. They recommend a full vaccination work up for a recently arrived migrant to Canada, including MMR, DTP for all adults as well as children with uncertain vaccination status, serological testing and vaccination in adults for varicella, and adding hepatitis B vaccination in specific adult and child migrant populations from high-burden countries. The IOM has also recommended vaccination for recently arrived migrants/refugees according the national schedule for the country, with priority for protection against measles, rubella, diphtheria, tetanus, pertussis, polio, Hib and hepatitis B [17]. Whilst such guidance may be informative for harmonising approaches to vaccination in migrants, our data suggest that there remains a critical need now to generate a comprehensive set of guidelines for the EU/EEA context and – importantly – work towards uptake and implementation of guidance at the national level across the EU/EEA targeting both child and adult migrants in catch-up vaccination. This aligns with priorities of the WHO European Vaccine Action Plan [10], that seek to reduce inequities in access to vaccination in migrant populations in Europe. Strong promotional campaigns and a commitment to improving access to primary care – whilst being mindful of the different experiences that each EU country has with respect to migration demographics and health-care resources – will be crucial for improving the health status of recently arrived migrants across Europe.

**Table 2:** Summary of recommendations from the Canadian guidelines [37].

Vaccine-preventable disease	Children <18 years	Adults >18 years
Measles, mumps, rubella	Vaccinate all migrant children with missing or uncertain vaccination records using age appropriate vaccination	Vaccinate all adults without immunisation record
Diphtheria, pertussis, tetanus, polio	Vaccinate all migrant children with missing or uncertain vaccination records using age appropriate vaccination	Vaccinate all adult migrants without immunisation records
Varicella	Vaccinate all migrant children <13 years with varicella vaccine without prior serological testing	Screen all migrants from tropical countries of 13 years and older for serum varicella antibodies, and vaccinate those found to be susceptible
Hepatitis B	Screen children where seroprevalence of is >2%. Vaccinate those who are susceptible	Screen adults where seroprevalance of is >2%. Vaccinate those who are susceptible
Tuberculosis	Screen children, adolescent <20 years of age from countries with high incidence as soon as possible after their arrival with a tuberculin skin test. If positive, rule out active tuberculosis and then treat latent tuberculosis infection.	Screen those 20–50 year of age from countries with high incidence as soon as possible after their arrival with a tuberculin skin test. If positive, rule out active tuberculosis and then treat latent tuberculosis infection.

## Conflicts of interest

The authors report that they have nothing to declare.

## Funding

This research was funded by the European Society of Clinical Microbiology and Infectious Diseases through the ESCMID Study Group for Infections in Travellers and Migrants (ESGITM). LBN, SH, and JSF receive funding from the UK National Institute for Health Research Imperial Biomedical Research Centre, the Imperial College Healthcare Charity, and the Wellcome Trust (Grant number 209993/Z/17/Z).

## Key points

∗ There is striking variation in policies relating to the vaccination of recently arrived migrants across Europe. Six (18.8%) countries had comprehensive guidance and regulations specific to the vaccination of migrants, of which in 2 countries guidance only focused on child migrants. It is not clear to what extent guidelines are applied in practice.
∗ There is heterogeneity in approaches to priority vaccination in child, adolescent, and adult migrants. Polio is the most commonly administered vaccine and other vaccinations according to guidelines analysed – for example hepatitis B – may not be considered.
∗ Differences were found across countries when migrant presented with missing or incomplete vaccination records and a lack of clarity in terms of how to approach catch-up vaccination
∗ There is a lack of clarity on optimum approaches to vaccination in migrants, and a need for robust research and data collection in this area to explore and assess what works best in terms of the implementation of vaccination strategies in both child and adult migrants.
∗ More emphasis must be placed on ensuring migrant-specific guidance is disseminated to front-line healthcare professionals to improve vaccine delivery and uptake in diverse migration populations across the region.
